# Age-Friendly Health Care: A Systematic Review

**DOI:** 10.3390/healthcare9010083

**Published:** 2021-01-16

**Authors:** Jéssica Tavares, Gonçalo Santinha, Nelson P. Rocha

**Affiliations:** 1 GOVCOPP, Department of Social, Political and Territorial Sciences, University of Aveiro, 3810-193 Aveiro, Portugal; g.santinha@ua.pt; 2 IEETA, Department of Medical Sciences, University of Aveiro, 3810-193 Aveiro, Portugal; npr@ua.pt

**Keywords:** health care providers, age-friendly principles, education and training, management health system, physical environment

## Abstract

Background: Health care provided to older adults must take into account the characteristics of chronic diseases and the comorbidities resulting from ageing. However, health services are still too oriented towards acute situations. To overcome this problem, the World Health Organization (WHO) proposed a set of Age-Friendly Principles that seek to optimize the provision of health care for this population. This article aims to understand how such Principles are considered in the implementation of age-friendly health care worldwide. Methods: A systematic review was conducted to synthesize the literature on age-friendly health care in accordance with the PRISMA recommendations in the PubMed, Web of Science, and Scopus databases. Results: The research identified 34 articles, with only seven recognizing the WHO Principles and only four using the implementation toolkit. In addition, in the context of primary care, three studies recognize the WHO Principles, but only two use the toolkit. Conclusions: The WHO Principles are being implemented in health care, but in a smaller scale than desired, which reveals possible flaws in their dissemination and standardization. Thus, a greater scientific investment in age-friendly health care should be considered, which represents a greater operationalization of the Principles and an evaluation of their effectiveness and impacts.

## 1. Introduction

Population ageing is a demographic phenomenon worldwide. It echoes in the main causes of population morbidity and mortality, since the prevalence of chronic and degenerative diseases increases with age [1]. In fact, the increase in the demand for health care services by the older adults is indisputable, given that chronic diseases require long-term treatments, and slow and complicated recoveries [2,3].

Health care services are, however, poorly organized to provide care to the older adults with complex comorbidities and weaknesses [4]. Historically, health care services have been designed to optimize care for a younger population that requires rapid diagnosis and medical or surgical responses to episodes of acute illness [5]. Accordingly, the challenges that older adults already face in terms of declining health conditions, autonomy, and independence [6], may worsen due to adverse events in the health care environment, delirium [7], immobility [8], falls [9], pressure ulcers [10], and malnutrition [11], which can extend the care period and compromise the return to life in community [5,12].

WHO’s work in the past decade to develop age-friendly environments [13] aligns closely with efforts to adapt the structures and services to be accessible and inclusive for older people with varying needs and capacities [14], and accordingly has encouraged the need to redesign the health care supply [15].

The first contribution to this reorganization of health care services, however, appeared in Canada in 1999, with an approach to the hospital care called “Elder-Friendly Hospital” [16]. According to the authors, this form of care implies recognizing the risk of adverse consequences for the older adults during the hospital admission, and predicting and preventing solvable problems that can occur when the hospital provides health care to older adults. 

Years later, in 2004, WHO launched the “Age-friendly Primary Health Care” project [17] aiming to improve older adults’ care, participation, independence and dignity. Within this context, WHO defined three Age-Friendly Principles: (1) Information, Education, Communication, and Training, including staff training in clinical geriatrics and approaches to patient education; (2) Health Care Management Systems, i.e., adapting procedures, such as registration, to the special needs of older individuals and supporting continuity of care through updated medical records available at each visit; and (3) The Physical Environment, i.e., clean and comfortable centers that apply, as far as possible, the guidelines of universal design [18]. The implementation of the WHO Principles is supported by a toolkit developed by WHO in 2008, also in the context of primary health care [19]. The choice of primary care is justified by the fact that it is the first contact point that users, including older adults, establish with the Health System when they have a non-urgent health problem. Nevertheless, these Principles have a quite broad scope, and they can be used in addition to primary health care, as suggested by the International Network of Health Promoting Hospitals and Health Services [20]. In this frame, the idea is to generalize them to secondary care, which concerns health care provided by specialists in the hospitals, by referral from primary care or in emergent situations, and in terms of tertiary care, which provides services to hospitalized patients who require advanced medical treatment when referred by primary and secondary care.

On the other hand, the SARS-CoV-2 pandemic has enforced a rapid and unsettling transformation of the world healthcare systems. Detailed recommendations and reports have been published at international level particularly regarding the diagnosis and management of COVID-19 in the older adults [21]. However, little has been proposed for an appropriate response to older, frail, and multimorbid patients in different settings of care (long-term care, nursing homes, primary care, and hospitals) and for the management of geriatric syndromes (delirium, malnutrition, and falls) [22]. Due to its importance for providing the appropriate care to older patients, it is believed that the WHO Principles should be amongst health systems’ priorities especially when facing such pandemic [23].

Although the age-friendly health care concept has been addressed for more than a decade, it is still not clear the way and degree it is being implemented and the results achieved. Accordingly, the aim of this article is to synthesize the relevant academic literature concerned with the application of the WHO Principles on the implementation of age-friendly health care services worldwide. In this way, it is possible to draw conclusions about the nature of health care considered, the relevant areas of activity, and the degree of conformance with the WHO Principles. 

The reminder of the article is organized as follows. The next section details the methodological approach, in which research questions are introduced and the methods used are described. Then, results are presented: firstly, a general characterization of the studies is made and, secondly, the three distinct components (i.e., nature of care, fields of action, and level of conformance) that seek to answer the research questions are analyzed. Finally, a discussion of the results achieved is carried out, complemented by the presentation of some conclusions and limitations.

## 2. Materials and Methods

According to Egger, Davey-Smith, and Altman [24], systematic reviews aim to gather, critically assess, and conduct a synthesis of the results of multiple primary studies, allowing to create and organize the body of knowledge on a given topic. In addition, it seeks to answer clearly formulated questions, using systematic and explicit methods to identify, assess and interpret all the available and relevant research for the subject under study [25]. 

This systematic review followed the Preferred Reporting Items for Systematic Reviews and Meta-Analyses (PRISMA) guidelines [26]. In order to achieve the specific objective of this systematic review, i.e., to understand how the WHO Principles have been considered worldwide, the following research questions were formulated:

RQ 1: How are the Principles defined by WHO being implemented in primary health care?

RQ 2: How are the WHO Principles being implemented at the different levels of health care provision, even though their formulation focuses on primary care? 

RQ 3: What fields of action (i.e., the dimensions used in the adequacy of health care providers to older adults) are considered in the implementation of age-friendly health care?

The search terms used were age-friendly, elder-friendly, senior-friendly, healthcare, health care, primary health care, secondary health care, hospitals, health system, and health services. Two multidisciplinary databases, Scopus and Web of Science, were used in the search, as well as a specific database, PubMed (i.e., health care). In this last database, MeSH terms were included.

As inclusion criteria, all articles that addressed age-friendly health care were considered, with the respective publications in scientific journals, annals of conferences, or book chapters. The bibliographic research focused on all articles published since 2004, the publication date of the WHO Principles, until the end of 2019.

All articles not published in English, without abstract, without author, and without access to the full text were excluded. In addition, systematic reviews, patents, books, editorials, and prefaces were also excluded, as well as all articles that did not address the concept under analysis or were not relevant to the specific purpose of this review.

Data extraction and analysis involved a five-phase process. Firstly, we completed a title and abstract review for each search to determine topic and content relevance. Secondly, we constructed a data extraction tool based on the inclusion and exclusion criteria. In this phase, two of the authors read the full articles and independently selected and evaluated the studies. Thirdly, we contrasted and compared our independent decisions to arrive at consensus on included studies and the relevance of the information collected from those studies. In the fourth phase, a third author independently reviewed and analyzed the accuracy of the thematic content. Finally, the full team discussed and through consensus arrived at the final analysis of central themes: (i) nature of care, (ii) level of conformance, and (iii) fields of action. 

The first theme concerns the analysis of the nature of care related to the implementation of the WHO Principles. Although these Principles focus mostly on primary health care, it is expected that their application becomes widespread to the remaining levels of care. For this, it sought to identify the level of care providers where the studies were developed.

The second main theme explores the fields on which the institutions focus their actions to adapt health care to the older adults. In particular, and admitting the Principles proposed by WHO, it sought to understand how the fields identified in the articles are consistent with those of WHO. For this purpose, the existing definitions of each field identified in the articles were presented and correspondence with the WHO Principles was established. The relationship established can be observed in the Appendix A (Table A1).

The last theme concerns the detailed analysis of the level of conformance with the WHO Principles defined in the 2004 project and in the toolkit launched in 2008. In this context, the articles that recognize the WHO Principles and that use the implementation aid toolkit, as well as the respective study objectives, were identified.

## 3. Results

### 3.1. Literature Search 

**Figure 1:**
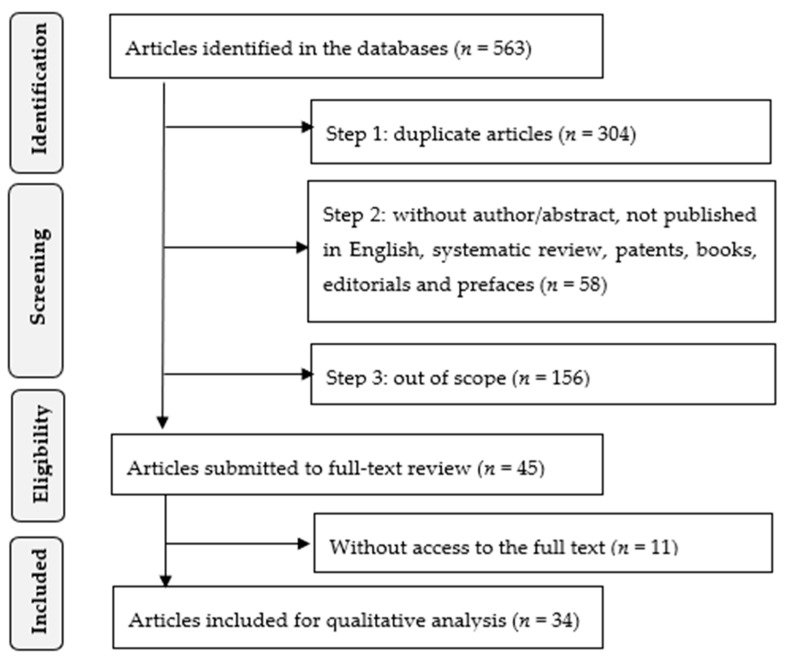
Flowchart of systematic review.

Figure 1 shows the PRISMA flowchart of this systematic review.

The initial search in the databases gathered a total of 563 articles, 215 from Scopus, 181 from Web of Science, and 167 from Pubmed (i.e., identification phase).

The initial screening phase (i.e., step 1) produced 259 articles after removing duplicates (*n* = 304 articles). 

Then, based on the titles and abstracts of the articles (i.e., step 2), a total of 201 studies were gathered as 58 articles were removed for the following reasons: (i) articles with no author and no abstract (*n* = 20 articles); (ii) articles not published in English (*n* = 14 articles); (iii) systematic literature review articles (*n* = 17 articles); and (iv) patents, books, editorials, prefaces, and official documents (*n* = 7 articles).

The remaining articles were analyzed, and it was concluded that only 45 were in the scope of age-friendly health care, with a total of 156 articles removed (i.e., step 3). Most of the excluded articles were related to health care provided to older adults, but unrelated to the “age-friendly” concept (*n* = 98 articles). Other large portion of eliminated studies, though addressing that concept, did it in the context of “age-friendly cities” [13] (*n* = 42 articles), hence, out of the scope of this study. The rest of the removed articles referred to the ageing process but did not present any specifications for health care or age-friendly environments (*n* = 16 articles). 

Considering the 45 eligible studies, it was not possible to access the full-text in eleven of them and, therefore, they have been excluded (i.e., eligibility phase). Contact with the authors was attempted to request full access to the articles, but no response was received.

An overview of the 34 included studies can be analyzed in the Appendix B (Table A2).

### 3.2. Characterization of the Studies

**Figure 2:**
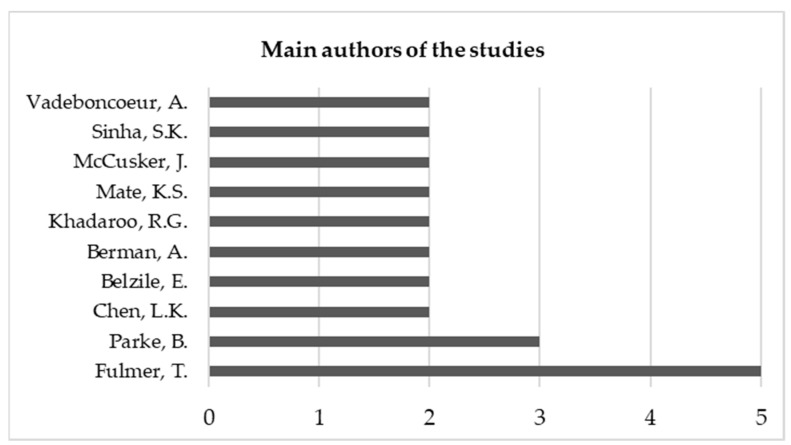
Number of publications by the main authors of the studies.

Considering the 34 studies under analysis, the authors who published the most were Fulmer and Parke, with five and three articles published, respectively. It should be noted that only one of the most cited articles was developed by one of the main authors (Parke with 26 citations in Scopus). Figure 2 shows the number of publications by author with more than one study published.

**Figure 3:**
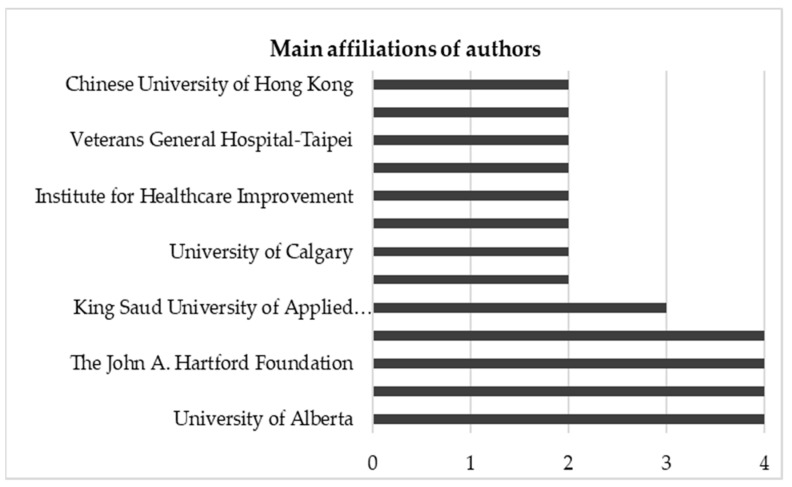
Number of publications by institutions affiliated with more than one author.

As for the authors’ affiliation, there was a higher incidence associated with the University of Alberta, the University of Taiwan, the University of Toronto, and the John A. Hartford Foundation, with four authors each. Figure 3 shows the number of publications by institution, with more than one author. In turn, the profile of the authors indicated that the vast majority of the studies involved health care providers and researchers (*n* = 15 articles), followed by studies developed exclusively by researchers (*n* = 14 articles) and, finally, articles written only by health care providers (*n* = 5 articles).

The subsequent continental distribution of the studies allowed to verify that there is a greater scientific production from America (*n* = 18 articles) and Asia (*n* = 11 articles), followed by Europe (*n* = 4 articles) and Africa (i.e., one article). Confirming this analysis, there is a greater number of articles from Canada (*n* = 11 articles), USA (*n* = 6 articles), and Taiwan (*n* = 3 articles), followed by South Korea, Hong Kong, and the Netherlands (*n* = 2 articles each) and, finally, Saudi Arabia, India, Iran, Turkey, Uganda, Brazil, Spain, and Portugal (*n* = 1 article each). 

**Figure 4:**
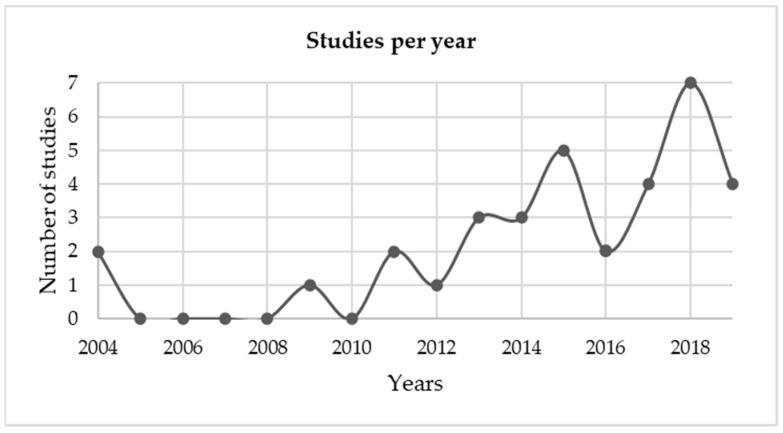
Number of publications per year.

Given the novelty of the theme, 2018 presented a peak in publications in the area (*n* = 7 articles), followed by 2015 (*n* = 5 articles). Figure 4 shows the number of publications per year. 

### 3.3. Nature of Care

Most of the 34 included studies were developed within the scope of secondary care (*n* = 23 articles). In this case, 13 articles focused only on a hospital department, namely, (i) Emergency Department, seven articles [27,28,29,30,31,32,33]; (ii) Surgery Department, two articles [34,35]; (iii) Acute Care Service, two articles [36,37]; (iv) Outpatient Department, one article [38]; and (v) Internal Medicine Department, one article [39]. The remaining ten articles analyze the entire hospital [5,40,41,42,43,44,45,46,47,48]. 

**Figure 5:**
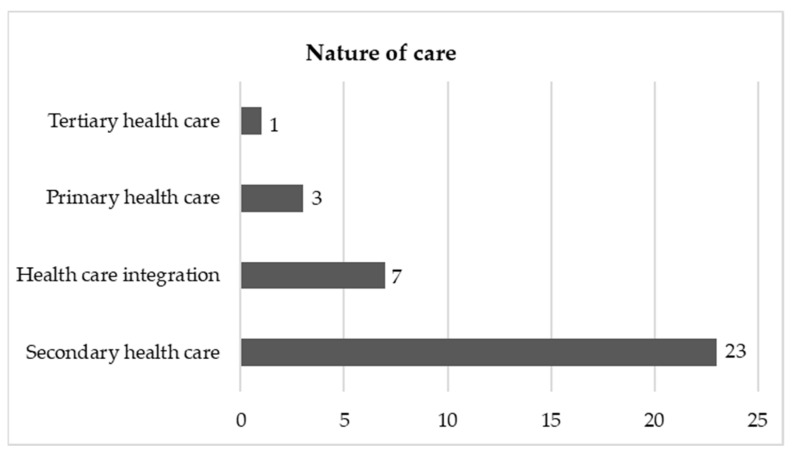
Number of publications by main types of health care provision.

As for other types of health care provision, there are three articles on primary care [49,50,51], one on tertiary care [52], and seven articles that address care integration [53,54,55,56,57,58,59]. It is understood that care integration concerns the promotion of integrated and continuous health care processes in space and time [60]. Figure 5 shows the distribution of studies according to the nature of health care provision.

### 3.4. Fields of Action

Only 19 studies detail the key attributes considered in the implementation of age-friendly health care. The remaining 15 articles discuss health care oriented to older adults, but do not define fields of action and/or of analysis. It should be noted that two articles, although identifying four fields, are not liable to be interpreted in light of the WHO Principles as they follow their own definition, proposed by the John A. Harford Foundation [55,56]. This organization proposed the concept of “Age-Friendly Health System” in 2016, defining an evidence-based geriatric care model based on four elements: mentation, medications, mobility, and matters most. The “mentation” element comprises the prevention, identification, and treatment of dementia, depression and delirium acquired in the services. The medication corresponds to the use of age-friendly drugs, which do not interfere with mobility or cognition. “Mobility” ensures that older adults move safely within the services to maintain their function. Finally, “matters most” concerns the recognition and alignment of health care with the goals and preferences of each older adult. 

Regarding the source of the fields in the 19 studies considered, only two articles use exactly the Principles proposed by WHO [40,51]. In turn, three articles define new fields based on adaptations to WHO’s Principles [44,47,49]; eight articles establish the fields identified grounded in relevant studies in the area [5,27,29,33,39,42,43,48]; one article establishes the fields based on WHO and on relevant studies [41]; and, finally, three articles do not explain the source [31,32,52]. 

In this sense, there was a prevalence of a set of four fields related to the concept of Age-Friendly Hospital—Management policy, Communication, and services, Care processes and Physical Environment—and a set of fields concerning the “Age-Friendly Emergency Department”—Social climate, Policies and procedures, Systems and processes of care, and Physical environment (See Table 1). 

The most common WHO Principle to these studies (with the exception of [58]) was the “Physical environment”, which required the attention of all authors in an exclusive or complementary manner with regard to the application of the guidelines of universal design. 

In general, it is possible to state that most of the studies analyzed in this category considered the three WHO Principles, even though they use different terminologies (compare with Table 1). Nevertheless, innovative fields arise in the scope of organizational leadership, ethics in care, religion, and availability of technical equipment (see Table 1).

### 3.5. Level of Conformance

In this phase of analysis, three different classes were identified. Classified as first stage, seven articles recognize and allude to the WHO project, but they do not use the toolkit in the development of their studies. The articles of Chiou and Chen [41], O’Keeffe [43], Kuo and Id [44], Rashmi et al. [47], and Kim et al. [48] start from the WHO’s main objective and are developed at the secondary health care level, seeking to implement and evaluate the concept of Age-Friendly Hospital through the identification of strategies and scales of assessment. It should be noted that the study of O’Keeffe [43] presents only the strategies in terms of the physical environment. As for Kanevetci and Yaman’s [50], their article addresses the WHO project and identifies the prevalent health problems to older adults in the context of primary health care, with no place for implementing the Principles. Fulmer’s [53] article, on the other hand, reflects the perspective of the integration of care, where the concept of the Age-Friendly Health System is addressed as a tool to protect older adults from abuse.

Classified as second class of conformance, four articles mention the WHO project and use the respective toolkit throughout the study. The articles of Ahmadi et al. [40], Woo et al. [51], and Ssensamba et al. [58] assess the quality of the services provided to the older adult population by conducting interviews and focus groups to staff and administrators, in order to identify the gaps and changes necessary to provide age-friendly health care (Age-Friendly Hospital, Age-Friendly Primary Health Care, and Age-Friendly Health System, respectively). In turn, Alhamdan et al. [49] assess the services provided to older adults in primary health care and the ease of use of those services by older adults based on the guidelines of the WHO toolkit regarding two Principles: management systems and physical environment.

The third stage category includes 23 articles which do not address the WHO guidelines, although they demonstrate concern for implementing age-friendly health care. In this set, there are articles concerning secondary health care in a context of care integration. On the one hand, there are studies in which an assessment of the current services provided to older adults is carried out, aiming to a future implementation of age-friendly health care [5,27,29,34,38,39], or in an attempt to formulate strategies that allow this implementation [30,33,42,45]. On the other hand, there are studies that have already implemented age-friendly health care and are concerned with measuring the results achieved [28,35,36,52].

In addition, there is the development of scales that allow the assessment of the changes implemented in providers [31,32], with the respective post-implementation assessment [52]. Furthermore, specific programs for improving health care in pre-determined hospital services are developed [37,46], with post-implementation assessment [28,35,36]. Finally, the need for services to adapt to older adults in a context of demographic and epidemiological changes is discussed [59] and the concept of Age-Friendly Health System is addressed [53,54,55,56,57].

## 4. Discussion

The first two research questions focused on the extent of the implementation level of the WHO Principles in different health care providers. The systematic review carried out allows us to state that such level is still quite low. Only seven out of 34 studies recognize the WHO project and only four of them use the implementation assistance toolkit. It is important to note that the three studies considered in the context of primary health care mention the WHO project, but only two use the toolkit.

Such findings may have three possible explanations. First, the WHO guidelines focus mostly on primary health care, making it hard to extrapolate to other levels of care, which address different needs. As such, the implementation at the level of secondary and tertiary care becomes reduced and ineffective due to the lack of official guidelines, which may justify the small number of studies that recognize the WHO toolkit and Principles. Still, the reduced number of studies on primary care underlines the overall limited range and dissemination of the guidelines issued by WHO.

Second, the exiting obstacles to implement the WHO Principles can justify the numbers above. Some studies indicate difficulties in managing the staff, as there is a lack of human resources to fulfill the needs of older adults [30,34,59]. The WHO Principles propose a manager for each older adult and a greater follow-up by nurses, just to mention a couple of examples, which require a greater availability of staff. Indeed, the lack of human resources seems to slow down the implementation of these Principles. The financial issue also plays an important role here [59]. The lack of economic resources makes it difficult to adapt services to the WHO model, especially with regard to changes in the physical environment. Thus, it is also considered one of the barriers to its correct implementation.

Third, the lack of studies assessing the benefits and impacts generated by the implementation of the WHO Principles may also justify the low results achieved here. Although official guidelines for operationalization are found (e.g., toolkit), the absence of scientific data proving the positive and/or negative effects on the institutions that have gone through this process of change may discourage potential stakeholders. However, it is worth noting the four studies that already show some concern in assessing the benefits of implementing these Principles in health care. Some of the pointed-out benefits concern the reduction of the four main geriatric syndromes, namely, episodes of falls, delirium, immobility, and incontinence, as well as a decrease in hospital readmission rates.

In conclusion, it can be argued that the WHO Principles are being reproduced in primary health care with the help of the toolkit, but in a lower number than desired (i.e., RQ 1). On the contrary, there is greater concern about these Principles at the level of secondary care, which reflects an outreach beyond the level for which they were initially intended. The implementation of these Principles is even thought out for tertiary care and in a perspective of care integration, although it is still precarious (i.e., RQ 2).

Concerning the last research question, which seeks to identify the fields analyzed in the studies, it was concluded that only 19 out of 34 articles considered key attributes to implement age-friendly health care. From these, only two studies used exactly the Principles defined by WHO, which means that, although the authors may recognize WHO’s work, they choose fields from other sources that best fit their reality. Accordingly, one can infer that the WHO Principles are not robust enough to allow their wide use in implementations reported by the scientific literature of the area.

In this line of thought, there was an incidence of the following set of fields in the area of secondary care: management policies, communication and services, health care processes, and physical environment. Parke and Brand [45] proposed these fields for hospital setting in 1999, before the WHO document was published. Similarly, the set of fields “social climate, policies and procedures, systems and processes of care and physical environment”, defined by Parke and Friesen in 2007 for the Emergency Department [61], has been recurrent. Thus, it can be argued that the fields defined in Parke’s work (e.g., [16,45,61]) have influenced subsequent studies in the area.

Despite the above comment, all studies address the three Principles proposed by WHO (i.e., RQ 3). It was verified that a significant number of studies have been carried out on the physical environment, perhaps because it is the field where more precise guidelines can be found and because it is a less complex area of change.

Finally, the emergence of new fields not considered by WHO is highlighted. These cover issues that need to pay attention to the way they can influence health outcomes in older adults. In particular, they provide a broader implementation of age-friendly health care, not limited to the training of staff, changes in health care processes and physical environment, but considering a broader horizon concerning the adequacy of health care for older adults.

### Limitations

This review has some limitations that are worth to be noted. Firstly, there is the possibility of omission of some relevant studies in the area due to the exclusion of articles published in languages other than English. Still, in order to minimize this limitation as much as possible, thorough searches were carried out on different databases. Secondly, limitations can be pointed out related to the keywords and databases used in the searches. Thus, the selection of another set of keywords and databases, with different journals indexed, could have resulted in the inclusion of hitherto unknown articles. In addition, as most of the articles collected were published in indexed journals, relevant articles published in non-indexed journals, or proceedings may have been excluded from this study. Thirdly, “grey literature” was not included in this review which might be considered a gap, since it is assumed that there are many local projects that are not published as scientific articles but are described in reports with limited scope. Despite these limitations, in methodological terms, a thorough and rigorous review selection and data extraction were sought in order to make the results relevant for the study of the impact of the WHO Principles on the implementation of age-friendly health care.

## 5. Conclusions

The aim of this study was to understand the impact the WHO Principles on the implementation of age-friendly health care worldwide. After this review, it can be said that the WHO project is promising in terms of care oriented to the needs of the older adults, who are the main users of health care. However, the WHO Principles and toolkit have not been given the desired attention and have not had the expected effect. Failures in the dissemination of the project and in the standardization of the guidelines may be limiting their implementation worldwide. These WHO Principles have particular importance in a COVID-19 pandemic context, where older adults show higher risk and worse health conditions leading to an increase in demand and hospitalization. In fact, it should be considered a greater scientific investment in the area of age-friendly health care, resulting in greater operationalization of the WHO Principles as well as in research that assesses their effectiveness and impacts in different contexts.

For future research, it is suggested to assess the reasons for the high unawareness of the WHO project and the low use of the toolkit. In other words, to understand why public policies are not acknowledging the use of the Age-Friendly Principles in health care. In addition, considering the only four studies that measure results, it is proposed to understand how the implementation of these WHO Principles can be evaluated, by whom and through which tools. Finally, it would also be interesting to see how the COVID-19 pandemic, which affected the various Health Systems, can promote changes in the operationalization of these WHO Principles by health care providers.

## Figures and Tables

**Table 1:** Fields in the articles and possible correspondence with the WHO Principles.

First Author, Year	Domains	WHO Principles
Ahmadi, 2015 [40]	(i) Information and training, (ii) Management systems, and (iii) Physical environment and accessibility	1, 2, 3
Alhamdan, 2015 [49]	(i) Services and (ii) Environment	2, 3
Boltz, 2013 [27]	(i) Social climate, (ii) Policies and procedures, (iii) Systems and processes of care, and (iv) Physical design	1, 2, 3
Chiou, 2009 [41]	(i) Management policy, (ii) Communication and services, (iii) Care processes, and (iv) Physical environment	1, 2, 3
Huisman, 2018 [52]	(i) Design	3
Kelley, 2011 [29]	(i) Social climate, (ii) Policies and procedures, (iii) Health care system, and (iv) Physical environment	1, 2, 3
Kim D., 2014 [42]	(i) Design	3
Kim Y., 2017 [48]	(i) Management policy, (ii) Communication and services, (iii) Care processes, and (iv) Physical environment	1, 2, 3
Kuo, 2019 [44]	(i) Management policy, (ii) Communication and services, (iii) Care processes, and (iv) Physical environment	1, 2, 3
Mcclelland, 2015 [30]	(i) Staffing, (ii) Policies and procedures, (iii) Transitions of care, (iv) Education, (v) Quality improvement, and (vi) Equipment/supplies	1, 2, 3
McCusker, 2015 [31]	(i) Screening and assessment, (ii) Staffing, (iii) Discharge planning, (iv) Community services, (v) Care philosophy, (vi) Evaluation and monitoring, and (vii) Physical environment	1, 2, 3
McCusker, 2018 [32]	(i) Screening and assessment, (ii) Clinical protocols, (iii) Staffing, (iv) Discharge planning, (v) Continuing education, (vi) Quality assessment, and (vii) Physical environment	1, 2, 3
O’Keeffe, 2004 [43]	(i) Physical environment	3
Rashmi, 2016 [47]	(i) Medical care services, (ii) Impatient services, (iii) Accessibility, (iv) Physical environment, and (v) Spiritual environment ^a^	1, 2, 3
Santos, 2016 [33]	(i) Social climate, (ii) Policies and procedures, (iii) Systems and processes of care, and (iv) Physical environment	1, 2, 3
Ssensamba, 2019 [58]	(i) Leadership and governance ^a^, (ii) Health financing ^a^, (iii) Human resources, (iv), Geriatric care delivery, (vi) Health management information systems, and (vii) Commodities and equipment ^a^	1, 2
Tavares, 2017 [39]	(i) Service policies and (ii) Environment	2, 3
Wong, 2014 [5]	(i) Organizational support ^a^, (ii) Care processes, (iii) Emotional and behavioral environment ^a^, (iv) Ethics in clinical care and research ^a^, and (v) Physical environment	1, 2, 3
Woo, 2013 [51]	(i) Information, education, and training, (ii) Health care management systems and (iii) Physical environment	1, 2, 3

^a^ Does not fit the WHO Principles. 1—Information, Education, Communication and Training; 2—Health Care Management Systems; 3—Physical Environment.

## Data Availability

No new data were created or analyzed in this study. Data sharing is not applicable to this article.

## Appendix A

**Table A1:** Relationship established between the domains named in the articles and the WHO Principles.

WHO Principles	Article Domains
Information, Education, Communication and Training	(i) Staffing, (ii) Human resources, (iii) Continuing education, (iv) Education, (v) Information and training, (vi) Social climate, (vii) Communication and services, (viii) Health management information systems, and (ix) Information, education, and training
Health Care Management Systems	(i) Care processes, (ii) Screening and assessment, (iii) Clinical protocols, (iv) Discharge planning, (v) Quality assessment, (vi) Policies and procedures, (vii) Systems and processes of care, (viii) Community services, (ix) Care philosophy, (x) Evaluation and monitoring, (xi) Transitions of care, (xii) Quality improvement, (xii) Service policies, (xiv) Management policies, (xv) Medical care services, (xvi) Impatient services, (xvii) Services, (xviii) Geriatric care delivery, (xix) Management systems, and (xx) Health care system
Physical Environment	(i) Physical design, (ii) Accessibility, (iii) Equipment/supplies, (iv) Environment, (v) Physical environment, (vi) Physical environment and accessibility, and (vii) Design

## Appendix B

**Table A2:** Summary of the 34 studies included for qualitative analysis.

First Author, Year	Objective
Ahmadi, 2015 [40]	To examine the age compatibility of public hospitals in Iran.
Alhamdan, 2015 [49]	To evaluate the health care provided to older adults in primary health care and the simple use of these services.
Boltz, 2013 [27]	To describe, in the nurse’s opinion, what should be improved in the care of older adults in the emergency department.
Campmany, 2019 [28]	To develop a program to assist fragility in the emergency department.
Chiou, 2009 [41]	To describe the concept of an age-friendly hospital and present the structure defined in Taiwan.
Fulmer, 2018 [53]	To demonstrate how an age-friendly health system can reduce the abuse on older adults.
Fulmer, 2018 [54]	To explain how an age-friendly health system can benefit older adults with dementia.
Fulmer, 2018 [55]	To explain what motivated the John A. Hartford Foundation to create the “age-friendly health care system” initiative
Hanson, 2017 [34]	To assess barriers and facilitators for the implementation of age-friendly surgical interventions in urgent situations.
Huisman, 2018 [52]	To present a structural approach to long-term care facilities for older adults in fragile health conditions.
Isaacs, 2019 [56]	To present the initiative “age-friendly health systems” developed by the John A. Hartford Foundation.
Kanevetci, 2018 [50]	To assess the health needs of older adults cared for in primary health care.
Kelley, 2011 [29]	To evaluate the current healthcare practices provided to older adults in the emergency department, identifying the gaps to become an age-friendly caregiver.
Khadaroo, 2015 [35]	To realize the impact of age-friendly approaches on the surgical environment in older adults undergoing acute care surgeries.
Kim D., 2014 [42]	To explore perceptions about the design elements of an age-friendly hospital.
Kim Y., 2017 [48]	To develop a Korean age-friendly hospital structure.
Kuo Y., 2019 [44]	To implement an age-friendly hospital certification process and assess changes in professionals’ knowledge and attitudes.
Kuo R., 2011 [38]	To assess the quality of outpatient services for older adult patients.
Mate, 2018 [57]	To describe the process of creating the John A Hartford Foundation’s “age-friendly healthcare systems” initiative.
Mcclelland, 2015 [30]	To review nursing interventions in the management of older adults’ patients in the emergency department.
McCusker, 2015 [31]	To develop and validate a preliminary assessment scale for the age-friendly emergency service.
McCusker, 2018 [32]	To develop a comprehensive quality assessment tool for an age-friendly emergency service.
O’Keeffe, 2004 [43]	To present guidelines for an age-friendly environment based on a literature review.
Parke, 2004 [45]	To develop personalized strategies that should be implemented in age-friendly hospitals.
Persoon, 2015 [46]	To develop a questionnaire to measure the care that older adults receive at the hospital, the nurses’ attitudes, and the perceptions of older adults’ about said care.
Rashmi, 2016 [47]	To develop the criteria for an age-friendly hospital and assess its validity.
Santos, 2016 [33]	To identify and analyze the aspects necessary to provide age-friendly health care in the emergency department, from the nurses’ perspective.
Sinha, 2014 [36]	To provide guidance on how the age-friendly acute care service can be replicated throughout the hospital.
Ssensamba, 2019 [58]	To explore Uganda’s public health system’s readiness to offer age-friendly health care.
Tavares, 2017 [39]	To evaluate policies and environments that promote functionality and physical activity in a hospital environment.
Verma, 2017 [37]	To analyze the development of models and practices of age-friendly care in the acute care service.
Wong, 2014 [5]	To self-assess age-friendly hospitals, by identifying promising practices and initiatives to improve the quality of the system.
Woo, 2013 [59]	To identify the needs of older adults and understand how healthcare services are suited to those needs.
Woo, 2013 [51]	To assess the compatibility of primary health care with age.
